# An Optimised Aqueous Extract of Phenolic Compounds from Bitter Melon with High Antioxidant Capacity

**DOI:** 10.3390/antiox3040814

**Published:** 2014-12-02

**Authors:** Sing Pei Tan, Costas Stathopoulos, Sophie Parks, Paul Roach

**Affiliations:** 1 School of Environmental and Life Sciences, University of Newcastle, Ourimbah, NSW 2258, Australia; E-Mails: sophie.parks@dpi.nsw.gov.au (S.P.); paul.roach@newcastle.edu.au (P.R.); 2 Faculty of Bioscience Engineering, Ghent University Global Campus, Incheon 406-840, Korea; E-Mail: costas.stathopoulos@ghent.ac.kr; 3 Central Coast Primary Industries Centre, NSW Department of Primary Industries, Ourimbah, NSW 2258, Australia

**Keywords:** bitter melon, phenolic compounds, antioxidant capacity, aqueous extraction, organic solvents

## Abstract

Bitter melon (*Momordica charantia* L.) is a tropical fruit claimed to have medicinal properties associated with its content of phenolic compounds (TPC). The aim of the study was to compare water with several organic solvents (acetone, butanol, methanol and 80% ethanol) for its efficiency at extracting the TPC from freeze-dried bitter melon powder. The TPC of the extracts was measured using the Folin-Ciocalteu reagent and their antioxidant capacity (AC) was evaluated using three assays. Before optimisation, the TPC and AC of the aqueous extract were 63% and 20% lower, respectively, than for the best organic solvent, 80% ethanol. However, after optimising for temperature (80 °C), time (5 min), water-to-powder ratio (40:1 mL/g), particle size (1 mm) and the number of extractions of the same sample (1×), the TPC and the AC of the aqueous extract were equal or higher than for 80% ethanol. Furthermore, less solvent (40 mL water/g) and less time (5 min) were needed than was used for the 80% ethanol extract (100 mL/g for 1 h). Therefore, this study provides evidence to recommend the use of water as the solvent of choice for the extraction of the phenolic compounds and their associated antioxidant activities from bitter melon.

## 1. Introduction

Bitter melon (*Momordica charantia* L.) is a popular medicinal fruit particularly in Asia and Africa, where many varieties are grown. For example, bitter melon has been associated with anti-cancer [1], anti-microbial [2], anti-inflammatory [3] and anti-diabetic properties [4]. The potential medicinal values of the fruit have been linked to its content of phenolics and their antioxidant properties [4,5,6,7].

The phenolic structure is comprised of an aromatic group with one or more hydroxyl groups [8]. Owing to the hydroxyl groups, most phenolics in bitter melon are hydrophilic compounds, such as gallic acid, gentisic acid, catehcin, chlorogenic acid, epicatechin, vanillin acid, protocatechuic acid, *p*-coumaric acid, *o*-coumaric acid, and *t*-cinnamic acid [9,10].

Studies have reported that phenolics have potent antioxidant and free radical-scavenging activities [11,12]. Whole bitter melon (flesh, aril and seeds) has been shown to be a good source of phenolic compounds [9] and one study demonstrated that the flesh, aril and seeds all had very high antioxidant activity [9]. Another study suggested that the antioxidant and free radical scavenging properties in the extracts of bitter melon could be attributed to flavonoids and other phenolic compounds [11]. The results also revealed that, on a weight per weight basis, the bitter melon had better 2,2-diphenyl-1-picrylhydrazyl (DPPH) radical scavenging and ferric reducing antioxidant power (FRAP) than vitamin E [11].

The phenolic compounds extracted from plant matrices and their associated antioxidant activities are dependent on the parameters of the method used, including the solvent type and polarity, the temperature, the length of the extraction, the number of times a sample is extracted, and on the condition of the raw material, including its particle size [13,14]. To date, several solvents, including methanol [15], water [12] and the combination of ethanol and water [10], have been used for extracting phenolic compounds from bitter melon.

Although studies have reported that organic solvents can be more effective than water at extracting phenolic compounds from plant materials [16,17], water is a more desirable extractant for the food industry because it is non-toxic, environmentally friendly and inexpensive compared to organic solvents [18].

To our knowledge, no published study has comprehensively investigated and optimised the aqueous extraction of phenolic compounds and their associated antioxidant activities from bitter melon and compared the optimised aqueous extraction of these compounds with that achieved with organic solvents. Therefore, this study aimed to maximise the aqueous extraction of the phenolic compounds from freeze-dried and powdered bitter melon and to compare the optimised aqueous extract with that obtained with the best organic solvent from methanol, 80% ethanol, butanol and acetone.

The conditions investigated for the aqueous extraction of the phenolic compounds from the powdered bitter melon were temperature, time, water-to-powder ratio, powder particle size and the number of extractions of the same sample. The optimised aqueous extract was then compared to the best of the organic solvent extract in terms of total phenolic content (TPC) and antioxidant capacity (AC). The correlation between the TPC and the AC of the extracts was also determined.

## 2. Materials and Methods

### 2.1. Plant Material

Bitter melon (Moonlight variety) fruits (20 kg), grown in the Northern Territory (Darwin, NT, Australia), were purchased from the Sydney markets (Sydney, NSW, Australia) and frozen at −20 °C until used. The frozen bitter melons were cut into slices (~1 to 2 mm) and placed in liquid nitrogen before drying in a FD3 freeze dryer (Rietschle Thomas, Seven Hills, NSW, Australia) at −40 °C for 72 h at 2 × 10^−1^ mbar. After freeze-drying, samples were ground into a powder using a commercial blender (John Morris Scientific, Chatswood, NSW, Australia). The powders were mixed thoroughly and sorted into six particle sizes (<0.25, 0.5–0.25, 1–0.5, 1–2, 2–2.8 and >2.8 mm) by sieving through a series of EFL 2000 stainless steel sieves (Endecotts Limited, London, England). The powders were then kept in sealed containers at −20 °C until used.

### 2.2. Chemicals

Methanol and acetone were obtained from Merck (Kilsyth, VIC, Australia). Ethanol was purchased from Fronine (Taren Point, NSW, Australia). Hydrochloric acid (36%) was obtained from Ajax Finechem (North Ryde, NSW, Australia) and *n*-butanol was purchased from Swift Australia Chemical Supplier (Mulgrave, VIC, Australia). Folin-Ciocalteau (FC) reagent, 2,2′-azinobis-(3-ethylbenzothiozoline-6-sulfonic acid (ABTS), 2,2′-diphenyl-1-picrylhydrazyl (DPPH), sodium carbonate, sodium nitrite, aluminium chloride, sodium hydroxide, vanillin, sodium acetate trihydrate, acetic acid, 2,4,6-tripyridyl-*s*-triazine (TPTZ), ferric (III) chloride hexahydrate, sulphuric acid and standards (trolox, gallic acid, rutin and aecsin) were purchased from Sigma-Aldrich (Castle Hill, NSW, Australia).

### 2.3. Initial Solvent Extractions

**Figure 1:**
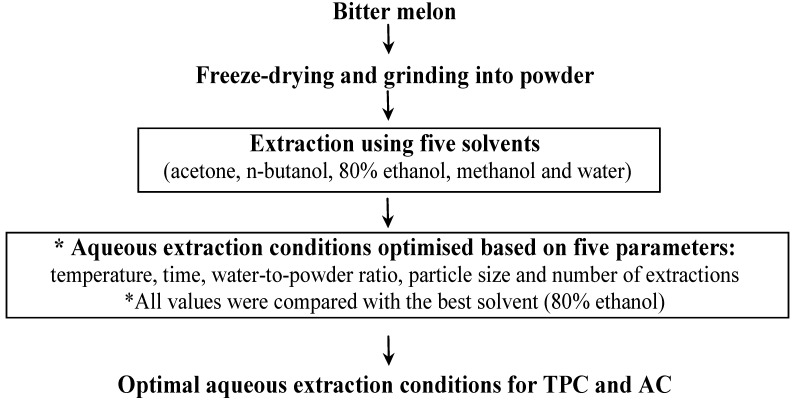
Diagram for the experimental design. Initially, the total phenolic content (TPC) and antioxidant capacity (AC) of the extracts of freeze-dried bitter melon powder obtained with five different solvents, including water, were compared. The aqueous extraction conditions were then optimised in terms of TPC and AC and compared to the 80% ethanol extract, which had the highest values in the initial solvent screening experiment.

The overall experimental design for the study is illustrated in Figure 1. Initially, extractions were done with five solvents: acetone, *n*-butanol, 80% ethanol (80 ethanol/20 water, v/v), methanol and deionised water. Bitter melon extracts were prepared by adding 1 g of freeze-dried bitter melon powder to 100 mL of solvent and extracting for 1 h using a shaking water bath (Ratek Instruments, Boronia, VIC, Australia). The extraction temperatures were set below the boiling point of each solvent: 50 °C for acetone, 60 °C for methanol and 80 °C for *n*-butanol, 80% ethanol and deionised water. After extraction, the samples were allowed to cool and settle on ice for 10 min. The extracts were then centrifuged at 4350× *g* for 10 min at 10 °C (Beckman Instruments Inc., Palo Alto, CA, USA) and the supernatant from each sample was filtered through a 0.45 μm syringe filter (Phenomenex, Pennants Hills, NSW, Australia) prior to analysis. All extractions were conducted in triplicate.

### 2.4. Optimising the Aqueous Extraction

The aqueous extraction was then optimised by testing, in sequential order, a range of temperatures, times, water-to-powder ratios, particle sizes and the number of times the same sample was extracted.

To determine the effect of the extraction temperature on the aqueous extraction of phenolic compounds, 1 g of freeze-dried bitter melon powder was extracted with 100 mL of deionised water at 5 °C and at intervals of 10° from 10 to 90 °C for 1 h using the shaking water bath.

The optimal temperatures for extraction (40 and 80 °C) were then used to determine the effect of the extraction time; 1 g of freeze-dried bitter melon powder was extracted with 100 mL of deionised water at 40 and 80 °C for 5, 10, 15, 20, 25, 30, 40, 50 and 60 min.

The optimal combination of time (5 min) and temperature (80 °C) was then used to determine the effect of the water-to-powder ratio; 1 g of freeze-dried powder was extracted in 10, 20, 25, 30, 40, 50 and 100 mL of deionised water.

To determine the effect of the freeze-dried particle size, the optimum conditions for temperature (80 °C), time (5 min) and water-to-powder ratio (40:1 mL/g) were used to extract 2.5 g of ground freeze-dried bitter melon having various sizes (<0.25, 0.25–0.50, 0.50–1.0, 1.0–2.0, 2.0–2.8 and >2.8 mm in diameter) with 100 mL of deionised water.

Finally, to evaluate the effect of extracting the same sample several times, 2.5 g of ground freeze-dried powder (1 mm) was extracted one, two or three times with 100 mL of deionised water at 80 °C for 5 min.

After each of the aqueous extraction experiments, the samples were allowed to cool on ice for 10 min before they were centrifuged and their supernatant was filtered through a 0.45 μm syringe filter (Phenomenex, Pennants Hills, NSW, Australia) prior to analysis.. All experiments were conducted in triplicate.

### 2.5. Extraction Efficiency

From the initial extractions done with the five solvents (acetone, *n*-butanol, 80% ethanol, methanol and deionised water), it was found that the 80% ethanol extract had the highest TPC and AC. Therefore, the 80% ethanol extract was chosen as the control extract for comparing the extraction efficiency for the various aqueous extractions—in terms of TPC and antioxidant capacity.

The extraction efficiency for the aqueous extractions, with respect to their TPC and AC, was calculated and expressed as a percentage of the 80% ethanol (EtOH) extract as follows:

Extraction efficiency % = (TPC or AC for aqueous extract/TPC or AC for EtOH extract) × 100.

#### 2.5.1. Total Phenolic Content

The TPC of samples was determined according to [19] with some modifications. Briefly, 300 μL of appropriately diluted bitter melon extract or standard solution (appropriate for each of the five extracting solvents, including a blank) was mixed and incubated with 300 μL of FC solution for 2 min before 2400 μL of a 50 g/L sodium carbonate solution was added. The solutions were mixed well and placed in the dark at room temperature for 2 h before the absorption was measured at 765 nm using a spectrophotometer (Carry 50 Bio, Varian Pty. Ltd., Mulgrave, VIC, Australia). Gallic acid was used as the standard and the TPC was expressed as mg gallic acid equivalents (GAE) per g of dry powder weight (mg GAE/g).

#### 2.5.2. ABTS Assay

The ABTS assay was conducted according Re *et al.* [20] with some modifications. Stock solutions of 7.4 mmol/L ABTS and 2.6 mmol/L potassium persulfate in deionised water were prepared and kept at 4 °C until used. Fresh working solution was prepared for each assay by mixing the two stock solutions in equal quantities and incubating for 12–16 h in the dark at room temperature. Then, 1 mL of the working solution was mixed with 60 mL methanol to obtain an absorbance of 1.1 ± 0.02 units at 734 nm. Each appropriately diluted bitter melon extract and standard solution (150 μL) was mixed and incubated with 2850 μL of the working solution for 2 h in the dark at room temperature before the absorption was measured at 734 nm. Trolox was used as a standard and the AC was expressed as μmol of trolox equivalents (TE) per g of dry powder weight (μmol TE/g).

#### 2.5.3. DPPH Assay

The DPPH assay was conducted according to Brand-Williams *et al.* [21] with some modifications. In brief, a stock solution of 0.6 mol/L DPPH in methanol was prepared and kept at −20 °C until used. Fresh working solution was prepared for each assay by mixing 10 mL of stock solution with 45 mL of methanol to obtain an absorbance of 1.1 ± 0.02 units at 515 nm. Each appropriately diluted bitter melon extract and standard solution (150 μL) was mixed and incubated with 2850 μL of working solution for 30 min at room temperature before the absorption was measured at 515 nm. Trolox was used as a standard and the AC was expressed as μmol trolox equivalents (TE) per g of dry powder weight (μmol TE/g).

#### 2.5.4. FRAP Assay

The FRAP assay was conducted according to Benzie and Strain [22] with some modifications. The stock solutions of 300 mmol/L acetate buffer pH 3.6, 10 mmol/L TPTZ in 40 mmol/L HCl and 20 mmol/L ferric (III) chloride hexahydrate were prepared and kept at 4 °C until used. Fresh working solution was prepared for each assay by mixing 100 mL of acetate buffer, 10 mL of TPTZ and 10 mL of ferric (III) chloride hexahydrate in a ratio of 10:1:1 and incubated at 37 °C before it was used. Each appropriately diluted bitter melon extract and standard solution (150 μL) was mixed and incubated with 2850 μL of the working solution for 30 min in the dark at room temperature before the absorption was measured at 593 nm. Trolox was used as a standard and AC was expressed as μmol trolox equivalents (TE) per g of dry powder weight (μmol TE/g).

### 2.6. Statistical Analysis

The Statistical Package for Social Science (SPSS) Version 19 (IBM Australia Limited, St Leonards, NSW, Australia) was used for data analyses. All experiments were conducted in triplicate and the one-way ANOVA and the Bonferroni *post-hoc* test were used to determine any significant differences between the mean values for the different treatments within an experiment. The Pearson correlation test was performed to establish the significance of correlations between the TPC and AC.

## 3. Results

### 3.1. Initial Solvent Extractions

**Figure 2:**
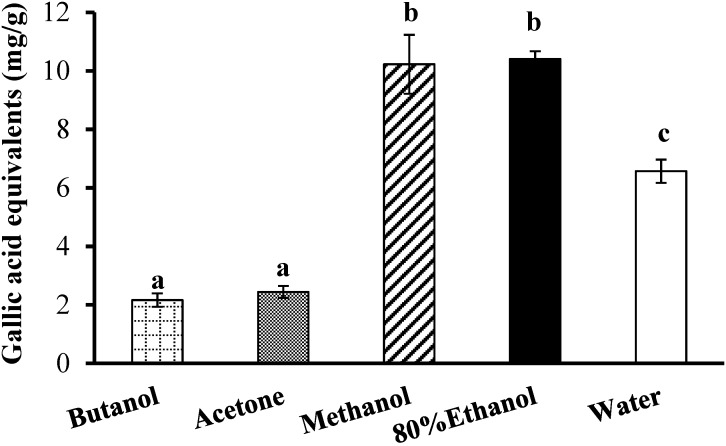
Total phenolic content of bitter melon extracts obtained with five solvents. Values are means ± standard deviations (*n* = 3) and those not sharing a superscript letter are significantly different (*p* < 0.05) from each other.

**Figure 3:**
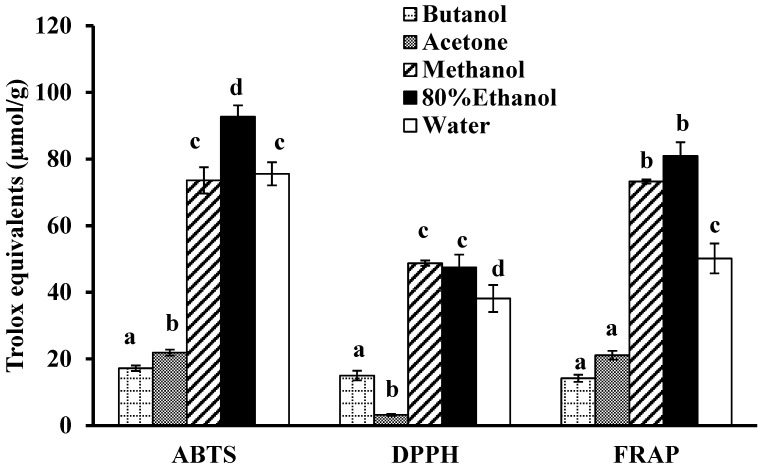
The 2,2′-azinobis-(3-ethylbenzothiozoline-6-sulfonic acid (ABTS), 2,2′-diphenyl-1-picrylhydrazyl (DPPH) and ferric reducing antioxidant power (FRAP) assays were used to determine the antioxidant capacity. Values are means ± standard deviations (*n* = 3) and those not sharing a superscript letter are significantly different (*p* < 0.05) from each other.

Initially, the freeze-dried bitter melon powder was extracted with five solvents: acetone, *n*-butanol, 80% ethanol, methanol and deionised water. The 80% ethanol extract had the highest TPC (Figure 2) and the highest AC in all three antioxidant assays (Figure 3). The methanol extract also exhibited the highest TPC (Figure 2) and the highest AC in the DPPH and FRAP antioxidant assays but had a significantly lower AC than the 80% ethanol extract in the ABTS assay (Figure 3). The extraction of phenolic compounds by water was significantly less effective than for 80% ethanol and methanol but was about three times more effective than for butanol and acetone.

The AC for the aqueous extract was also much higher than for the butanol and acetone extracts in all three antioxidant assays (Figure 3). There was no significant difference between the ABTS values for the aqueous and the methanol extracts but the AC for the aqueous extract was lower than for the 80% ethanol extract (Figure 3). In the DPPH and FRAP assays, the aqueous extract had a significantly lower AC than both the methanol and 80% ethanol extracts (Figure 3). Overall, strong correlations were observed between the TPC of the various solvent extracts (Figure 2) and their AC (Figure 3) as measured with the three assays: ABTS (*R* = 0.94), DPPH (*R* = 0.95) and FRAP (*R* = 0.99).

### 3.2. Optimising the Aqueous Extraction

**Figure 4:**
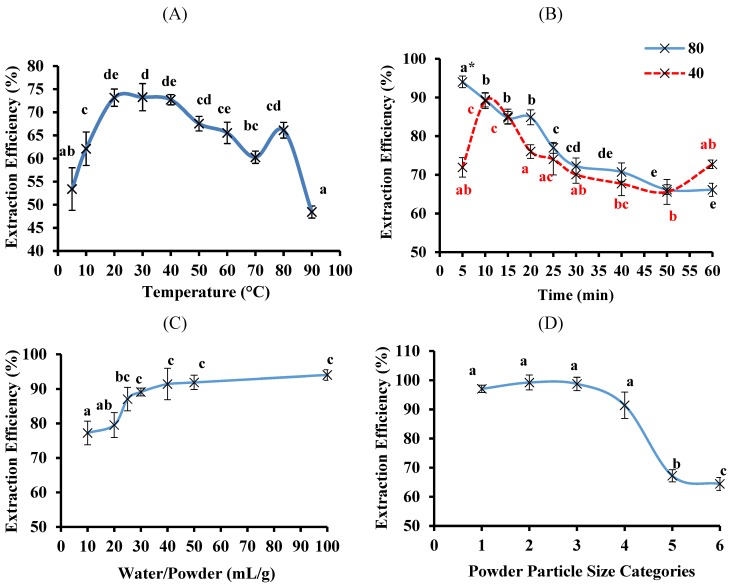

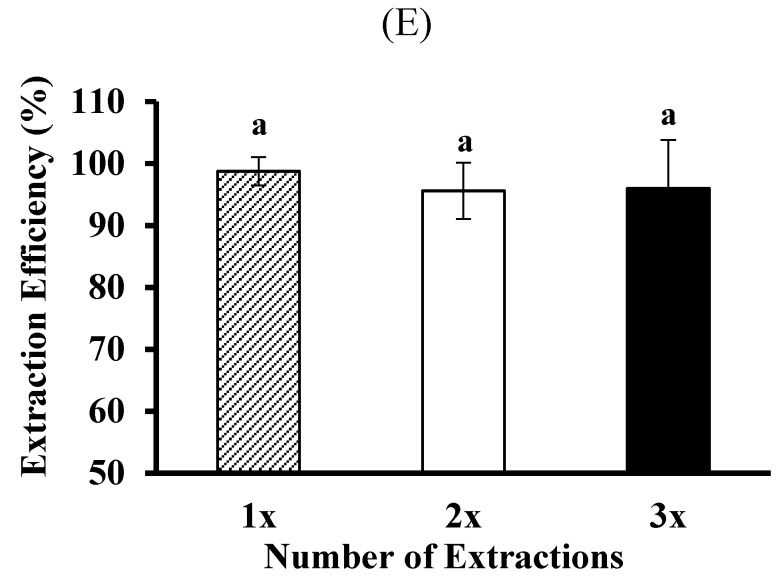
The extraction efficiency for phenolic compounds from bitter melon using water. The aqueous extraction was optimised using one-variable-at-a-time method and each variable was tested in sequential order: (**A**) temperature; (**B**) time; (**C**) water-to-powder ratio; (**D**) powder particle size and (**E**) number of times the same sample is extracted. The powder particle size categories were: (1) <0.25, (2) 0.25–0.05, (3) 0.5–1.0, (4) 1.0–2.0, (5) 2.0–2.8 and (6) >2.8 mm. The extraction efficiency was relative to the 80% ethanol extract and data are means ± standard deviations (*n* = 3) and those not sharing a letter are significantly different (*p* < 0.05) from each other. * This value is also significantly different (*p* < 0.05) from the values obtained at 40 °C.

**Figure 5:**
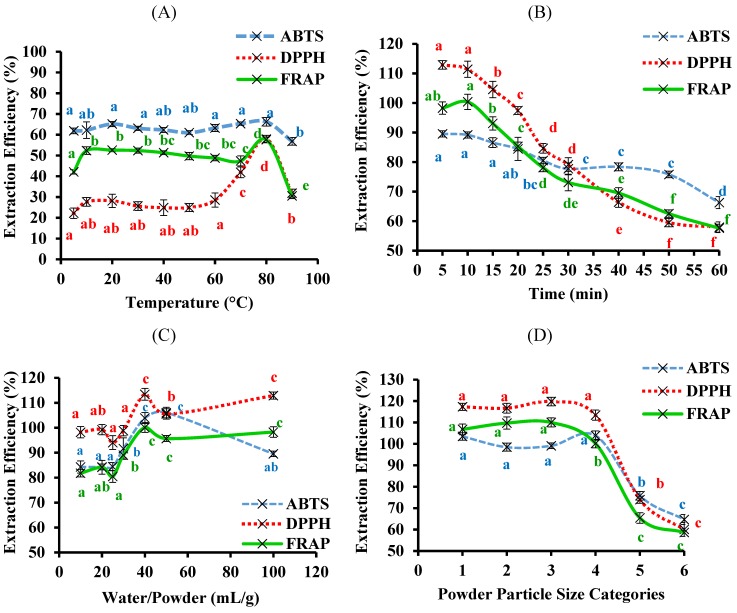

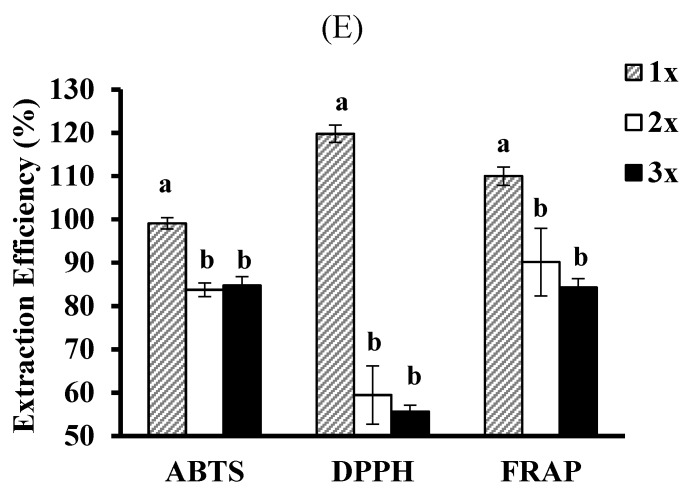
The extraction efficiency for antioxidant capacity from bitter melon using water. The aqueous extraction was optimised using one-variable-at-a-time method and each variable was tested in sequential order: (**A**) temperature; (**B**) time; (**C**) water-to-powder ratio; (**D**) powder particle size and (**E**) number of times the same sample is extracted. The powder particle size categories were: (1) <0.25, (2) 0.25–0.05, (3) 0.5–1.0, (4) 1.0–2.0, (5) 2.0–2.8 and (6) >2.8 mm. The extraction efficiency was relative to the 80% ethanol extract and data are means ± standard deviations (*n* = 3) and those not sharing a letter are significantly different (*p* < 0.05) from each other.

The optimal conditions for the aqueous extraction of the bitter melon phenolic compounds and their antioxidant activities were then determined by testing, in sequential order, a range of temperatures, times, water-to-powder ratios, particle sizes and the number of times the same sample was extracted. The results, expressed as extraction efficiency relative to the 80% ethanol extract, for TPC are presented in Figure 4 and those for AC in Figure 5.

The extraction efficiency for TPC was highest at temperatures between 20 and 50 °C and at 80 °C (Figure 4A). Therefore, the extraction time experiment was done at two temperatures, 40 and 80 °C, which showed that 80 °C for 5 min was the optimal combination of temperature and time (Figure 4B). Notably, the extraction efficiency for TPC obtained for 5 min at 80 °C was higher than the highest efficiency at 40 °C, which was obtained at 10 min (Figure 4B). Importantly, only 5 min was needed for the extraction, which is a much shorter time than the 1 h usually used for organic solvent extractions.

Using 80 °C for 5 min, it was then found that values between 25 and 100 mL/g for the water-to-powder ratio gave the highest extraction efficiency for TPC (Figure 4C). From these results, a water-to-powder ratio of 40 mL/g was chosen as being in the optimal range. Therefore, less water was needed than 100 mL/g, which is usually used for organic solvent extractions.

The extraction conditions of 80 °C for 5 min and a water-to-powder ratio of 40 mL/g were then used for testing the effect of powder particle size. As seen in Figure 4D, the extraction efficiency for TPC was higher at powder particle sizes ≤2.0 mm in diameter compared to sizes ≥2.0 mm. Therefore, a powder particle size of ≤1.0 mm was chosen as being in the optimal range.

Finally, the number of times the same powder same is extracted was tested using a powder particle diameter size of ≤1.0 mm with a water-to-powder ratio of 40 mL/g at 80 °C for 5 min. There was no significant difference in the extraction efficiency for TPC whether a sample for extracted once, twice or thrice (Figure 4E) and thus, a single step was considered optimal.

Similarly, for the AC of the aqueous extracts, the highest extraction efficiency was observed at 80 °C for the DPPH and FRAP assays (Figure 5A) while the extraction temperature had less of an effect on the AC measured using the ABTS assay. Therefore, these results supported the choice of 80 °C as the optimal temperature. Furthermore, doing the aqueous extraction at 80 °C for 5 min also gave optimal AC values (Figure 5B). Subsequently, a water-to-powder ratio of 40 mL/g (Figure 5C), a powder particle diameter size of ≤1.0 mm (Figure 5D) and one extraction (Figure 5D) proved to be optimal for the aqueous extraction done at 80 °C for 5 min, in terms of AC measured by the three antioxidant assays, the same optimal conditions as for TPC (Figure 4).

As seen in Table 1, the TPC and the AC of the aqueous extract were substantially improved under the optimised conditions compared to the values shown in Figure 2 and Figure 3, respectively. Importantly, this meant that the TPC and the AC (measured with the ABTS assay) of the optimised aqueous extract were also the same as for the 80% ethanol extract (Table 1). Furthermore, the AC of the aqueous extract was 20% and 10% higher for the DPPH and FRAP assays, respectively, compared to the 80% ethanol extract (Table 1).

**Table 1:** The phenolic compound content (TPC) and the antioxidant capacity of the optimised aqueous extract of bitter melon compared with the 80% ethanol extract.

Chemical Properties	Aqueous Extract	80% Ethanol Extract
TPC (mg GAE/g dry basis)	10.6 ± 0.2 ^a^	10.7 ± 0.3 ^a^
ABTS (μmol TE/g dry basis)	94.8 ± 1.3 ^a^	95.7 ± 3.5 ^a^
DPPH (μmol TE/g dry basis)	58.6 ± 1.0 ^a^	49.0 ± 4.0 ^b^
FRAP (μmol TE/g dry basis)	91.9 ± 1.8 ^a^	83.5 ± 4.3 ^b^

Values are means ± standard deviations (*n* = 3) and those in a row not sharing a superscript letter are significantly different from each other (*p* < 0.05).

Finally, the TPC of the various aqueous extracts was shown to have a high association with AC measured with the three antioxidant assays (Table 2). However, the association with TPC was 10% and 15% lower for the AC measured with the ABTS and the DPPH assays, respectively, compared to with the FRAP assay. This was despite the AC being highly correlated between the three assays (≥0.90).

**Table 2:** Pearson correlation coefficients (*R*) for the total phenolic content (TPC) of aqueous extracts and their total antioxidant capacity measured with the three assays.

Correlation	ABTS	DPPH	FRAP
TPC	0.830 **	0.784 **	0.922 **
FRAP	0.919 **	0.937 **	
DPPH	0.901 **		

** All correlations were significant at *p <* 0.01 (*n* = 132).

## 4. Discussion

This study demonstrated that the aqueous extraction of phenolic compounds from bitter melon was as efficient as organic solvent extractions, when the extraction conditions were optimised. In the present study, before optimisation, 80% ethanol was shown to be the most suitable solvent for extracting phenolics and their associated AC from bitter melon (Figure 2 and Figure 3). This finding is consistent with the study on phenolics and antioxidant activity of bitter melon by Horax *et al.* (2010), which demonstrated that a mixture of 80 ethanol/20 water (v/v) was the optimal ethanol: water solvent [10]. The TPC of the aqueous extract was 63% lower than for the best organic solvent, 80% ethanol (Figure 2), and the AC was at least 20% lower (Figure 3). However, after optimisation, the TPC and the AC of the aqueous extract were equal or higher than for 80% ethanol (Table 1). Furthermore, less solvent (40 mL water/g) and less time (5 min) were needed than was used for the 80% ethanol extract (100 mL/g for 1 h).

In general, low temperatures (5 and 10 °C) reduced the extraction of phenolic compounds from bitter melon whereas higher temperatures (20–80 °C) improved the extraction (Figure 4A and Figure 5A). Increasing the temperature of water is known to decrease its viscosity, which can increase the diffusion coefficients of solutes, including water soluble phenolic compounds, and thereby increase the extraction efficiency of the phenolic compounds from the bitter melon by the aqueous solvent [23]. Furthermore, heating allows the cell wall of plant materials to be more permeable, thereby allowing the solvent and the solutes, from inside the plant cell walls, to diffuse in and out of the plant cells more easily [18,24]. However, temperatures above 80 °C lowered the extraction efficiency; this may have reflected degradation of the phenolic compounds, which can occur at these high temperatures [25].

The length of the extraction at 80 °C also had a significant impact (Figure 4B and Figure 5B); interestingly, only 5 min was needed to reach the maximum for the extraction. This result indicates that the phenolic compounds in bitter melon may be highly soluble in water at 80 °C [26]. They are fairly polar compounds because, the polar organic solvents, 80% ethanol and methanol, were also more efficacious at extracting the phenolic compounds from bitter melon compared to the less polar solvents, acetone and butanol (Figure 2). Phenolic compounds have also been extracted from bitter melon in previous studies using polar solvents, including water [10,12].

Doing more than one extraction of the same sample also did not improve the extraction of the phenolic compounds or the antioxidant activity of the aqueous extract (Figure 4E and Figure 5E). This supports the suggestion that the predominant phenolic compounds in bitter melon are very hydrophilic; they were very efficiently extracted within 5 min during the first extraction. Other studies have also reported that the phenolic compounds were successfully extracted from bitter melon using water [11,12].

Although less likely, exposure of the phenolic compounds to temperatures higher than 80 °C (Figure 4A and Figure 5A) and for longer than 5 min at 80 °C (Figure 4B and Figure 5B) may have led to their degradation [25]. In the present study, great care was taken to reduce the length of time the extracts were exposed to the extraction temperature being tested; all samples were placed on ice immediately after the intended extraction time and allowed to cool down for 10 min. This is important because it has previously been noted that phenolic compounds in hot water extracts can substantially degrade when the extracts are left to cool down at room temperature [27].

The present finding on the length of the aqueous extraction are in agreement with a study on peanut skins [28] and on papaya leaves [29]. In contrast, the extraction efficiency for TPC and AC of olive seeds [30] and pomegranate leaves [31] extracts increased with longer extraction times. This suggests that the type of phenolic compounds in bitter melon are likely to be more like those in peanut skins and papaya leaves than in olive seeds and pomegranate leaves.

Increasing the water-to-powder ratio resulted in a higher extraction of the phenolic compounds (Figure 4C and Figure 5C), which was consistent with the extraction of water soluble components from plant material in general [32]. This is due to the high osmotic pressure, which results from the steep concentration gradient of the components in the plant material *vs.* the solvent that is generated at the high water-to-powder ratios [32]. The current finding was also in agreement with the results of studies on the extraction of other solutes from plant materials [33,34].

The powder particle sizes ≤2 mm, also resulted in higher extraction efficiencies than the powder sizes ≥2 mm (Figure 4D and Figure 5D). This was consistent with previous studies, which have shown that a smaller powder particle size results in a higher extraction efficiency of bioactive compounds from powdered plan material because the surface area of the powder in contact with the solvent is increased [18]. Furthermore, the diffusion distance within the powder particle is shorter and the concentration gradient is steeper between the plant material and the solvent, resulting in a higher extraction efficiency [35].

Finally, the TPC of the various aqueous extracts was highly correlated with the AC of the extracts as measured with the FRAP, DPPH and ABTS assays (Table 2). Similarly high correlations (*R* ≥ 0.94) were also observed between the TPC and the AC of the extracts in the original experiment with the five different solvents (Figure 2 and Figure 3). These findings are in agreement with a previous study on greenhouse-grown bitter melons of different varieties, which showed strong positive correlations between TPC and the AC measured with the three assays [12]. Therefore, these high correlations strongly suggest that the extracted phenolic compounds, including those in the aqueous extracts of bitter melon, possess potent antioxidant and free radical scavenging activities.

This study supports the use of water as the solvent of choice for the extraction of the phenolic compounds from bitter melon. However, whether the efficiency of extraction for phenolic compounds and their associated AC from bitter melon can be further improved using water with other emerging modern assisted-extraction technologies, such as ultrasonics [36], microwave [37], pulsed electric field [38], supercritical fluid [39] and pressurized liquid [40], needs to be investigated in future studies.

## 5. Conclusions

This study demonstrated that water could be used to effectively extract phenolic compounds and their associated antioxidant activities from bitter melon. It was shown that the aqueous method could be optimised to give an extraction equivalent to that obtained with the best organic solvent tested (80% ethanol) and less solvent and less time was required with water than with 80% ethanol. The optimal conditions for the aqueous extraction were a single extraction at 80 °C for 5 min at a water-to-powder ratio of 40:1 mL/g and a powder particle size of 1 mm. Therefore, this study provides evidence to recommend the use of water as the solvent of choice for the extraction of the phenolic compounds from bitter melon.
